# Tracking the impact of dietary quality scores on metabolic health: Insights from the Azar Cohort on patients with type 2 diabetes mellitus

**DOI:** 10.34172/hpp.025.44297

**Published:** 2025-07-15

**Authors:** Meysam Zarezadeh, Mehrdad Jamali, Elnaz Faramarzi, Parsa Jamilian, Nima Radkhah, Ahmad Saedisomeolia, Zohreh Ghoreyshi, Alireza Ostadrahimi

**Affiliations:** ^1^Faculty of Nutrition and Food Science, Tabriz University of Medical Sciences, Tabriz, Iran; ^2^Student Research Committee, Tabriz University of Medical Sciences, Tabriz, Iran; ^3^Liver and Gastrointestinal Diseases Research Center, Tabriz University of Medical Sciences, Tabriz, Iran; ^4^School of Medicine, Keele University, Staffordshire, UK; ^5^College of Health Sciences, Education Centre of Australia, Parramatta, NSW Australia; ^6^School of Human Nutrition, McGill University, 21111 Lakeshore, Ste. Anne de Bellevue, Montréal, QC H9X 3V9, Canada; ^7^Nutrition Research Center, Tabriz University of Medical Sciences, Tabriz, Iran

**Keywords:** Body mass index, Dietary inflammatory index, Glycemic control, Healthy eating index, Lipid profile, Prospective cohort, Type 2 diabetes mellitus

## Abstract

**Background:**

This study examined the association between changes in diet quality—assessed using the healthy eating index-2015 (HEI-2015) and the dietary inflammatory index (DII)—and lipid profiles and glycemic control in adults with type 2 diabetes.

**Methods:**

In this longitudinal study, data were collected from 103 adults with type 2 diabetes at two time points, six years apart (baseline and reassessment). The main predictors were changes in HEI-2015 and DII scores over time. The primary outcome measures were lipid profile components (LDL, HDL, total cholesterol, triglycerides) and glycemic control (FBS). Associations were examined using regression models adjusted for age, sex, body mass index (BMI), and energy intake.

**Results:**

No statistically significant associations were observed between HEI-2015 or DII scores and lipid or glycemic outcomes in the overall sample. However, subgroup analyses based on adjusted models revealed reduced odds of LDL elevation among individuals aged>60 (OR: 0.14, 95% CI: 0.02–0.91) and those with BMI≥30 (OR: 0.15, 95% CI: 0.02–0.90) in the highest tertile of DII change. These effects were not observed consistently across other subgroups.

**Conclusion:**

While no significant associations were found in the overall cohort, subgroup analyses revealed that individuals over 60 and those with BMI≥30 had reduced odds of LDL elevation with higher DII scores. These findings suggest potential population-specific effects of dietary inflammation on lipid metabolism. Despite limitations such as a small sample size and wide confidence intervals, this study provides valuable exploratory evidence and underscores the need for larger, targeted investigations to confirm whether anti-inflammatory diets can improve metabolic outcomes in high-risk subgroups.

## Introduction

 Diabetes mellitus is a chronic metabolic disorder marked by elevated blood glucose levels resulting from the body’s impaired ability to produce or effectively utilize insulin.^1^ Among its forms, type 2 diabetes is primarily driven by insulin resistance, which is influenced by genetic predisposition, obesity, sedentary behavior, unhealthy dietary patterns, and irregular sleep.^2^ The global prevalence of type 2 diabetes is escalating rapidly, with the number of cases projected to rise from 536.6 million in 2021 to 783.2 million by 2045.^3,4^ Therefore, poor disease management can lead to severe complications such as cardiovascular disease, kidney dysfunction, and neuropathy.

 Diet plays a central role in the management of type 2 diabetes. Excessive intake of sugars and refined carbohydrates destabilizes blood glucose levels, while inadequate physical activity and genetic factors exacerbate disease progression.^5,6^ A balanced and nutrient-rich diet has been shown to improve glycemic control, regulate lipid profiles—including low-density lipoprotein (LDL), high-density lipoprotein (HDL), triglycerides (TG), and total cholesterol—and reduce the risk of cardiovascular complications.^7^ In this context, diet quality indices have emerged as valuable tools for assessing nutritional adequacy, dietary variety, and moderation.^8^

 Two widely used indices are the healthy eating index (HEI) and the dietary inflammatory index (DII). These instruments evaluate adherence to dietary guidelines and the inflammatory potential of dietary patterns, respectively, and have been linked to metabolic outcomes in diabetes. For instance, Direktör et al found low HEI scores in patients with type 2 diabetes, highlighting the need for improved dietary practices.^9^ Similarly, Zeinalabedini et al reported that higher HEI adherence was associated with a 50% reduction in cardiovascular risk markers.^10^ Vahid et al observed that greater adherence to an adjusted HEI (AHEI) and anti-inflammatory diets correlated inversely with lipid levels, although their findings were not statistically significant.^11^

 Despite these findings, evidence on the relationship between diet quality and metabolic health remains inconsistent. Some studies report that high-quality dietary patterns, such as those measured by HEI-2015 and AHEI, are associated with reduced cardiovascular and metabolic risk

 Others, including Vitale et al, suggest no significant associations, while highlighting that pro-inflammatory diets, as assessed by the DII, may be linked to higher triglyceride and LDL levels and lower HDL cholesterol.^12^ These conflicting results may stem from differences in study design, population characteristics, dietary assessment methods, and confounding variables such as BMI, age, and medication use.

 Given these inconsistencies, further investigation remains important. Although the present study is limited by its modest sample size, it provides preliminary longitudinal evidence on the relationship between changes in diet quality—measured by HEI-2015 and DII—and lipid and glycemic outcomes in individuals with type 2 diabetes. By analyzing repeated measures over a six-year period and adjusting for key demographic and lifestyle factors, this study contributes exploratory insights that may inform future, larger-scale investigations aimed at clarifying the dietary determinants of metabolic health.

## Material and Methods

###  Study Design and Sample Size

 This prospective cohort study was conducted as part of the Azar cohort, a subset of the larger Prospective Epidemiological Research Studies in Iran (PERSIAN) based in Northwest Iran.^13,14^

 The studies protocol received ethical approval from the Tabriz University of Medical Sciences Ethics Committee (Approval ID: IR.TBZMED.REC.1400.882). Written informed consent was obtained from all participants or their legal guardians prior to enrollment.

 The objective was to assess associations between changes in diet quality, lipid profiles, and glycemic control over time. Data were collected during the baseline phase (2014–2017) and the follow-up phase (2020–2021). A total of 103 individuals with type 2 diabetes were selected based on predefined inclusion criteria. Demographic data—including age, gender, education level, and marital status—were collected using standardized forms. Additional details on the cohort’s design and methodology have been published elsewhere.^15^ Anthropometric and biochemical measurements, dietary assessments via food frequency questionnaire (FFQ), and diet quality scoring were conducted at both time points.

###  Inclusion and Exclusion Criteria

 Participants were eligible if they were aged 35–70 years, had a confirmed diagnosis of type 2 diabetes, and had completed both baseline and follow-up assessments. Required data included serum samples, cardiovascular risk assessments, and dietary intake via FFQ at both time points.

 Exclusion criteria included pregnancy or lactation, implausible energy intake reports (< 800 or > 5200 kcal/day), extreme BMI values (< 18.5 or > 46 kg/m^2^), and loss to follow-up.

###  Diabetes Definition 

 Diabetes status was determined based on physician diagnosis and participant self-report at the time of enrollment.

###  Biochemical Analysis

 Blood samples were collected in the morning following a 12-14 hour fast. Enzyme-based methods were employed to measure fasting blood sugar (FBS), serum TG, LDL, cholesterol, and HDL. Lipid profile classification and categorization were conducted in accordance with the Adult Treatment Panel III (ATP III) guidelines, which provide a structured framework for assessing cholesterol and triglyceride levels to evaluate cardiovascular risk. According to ATP III, LDL-C is the primary target for therapy, with levels categorized as optimal (< 100 mg/dL), near optimal (100–129 mg/dL), borderline high (130–159 mg/dL), high (160–189 mg/dL), and very high ( ≥ 190 mg/dL). Similarly, HDL-C, known for its protective role against heart disease, is considered low when below 40 mg/dL in men and 50 mg/dL in women, normal between 40–59 mg/dL, and high (protective) at ≥ 60 mg/dL. cholesterol is classified as desirable (< 200 mg/dL), borderline high (200–239 mg/dL), and high ( ≥ 240 mg/dL), while TG is categorized as normal (< 150 mg/dL), borderline high (150–199 mg/dL), high (200–499 mg/dL), and very high ( ≥ 500 mg/dL). Furthermore, ATP III emphasizes the importance of FBS in metabolic risk assessment, defining normal levels as < 100 mg/dL, impaired fasting glucose (prediabetes) as 100–125 mg/dL, and diabetes as ≥ 126 mg/dL. These guidelines serve as the foundation for evaluating lipid abnormalities and determining appropriate lifestyle modifications and pharmacological interventions to reduce the risk of cardiovascular disease.^16^

###  Dietary Intake

 Participants’ dietary intakes were evaluated and analyzed as part of a validated study conducted by Eghtesad et al.^17^ The assessment utilized a 168-item FFQ, which was specifically validated for the Iranian population to ensure cultural and dietary relevance.^17,18^ This tool provided comprehensive insights into the participants’ dietary habits, allowing for accurate measurement of nutrient intake and its potential association with health outcomes. The rigorous validation of the FFQ in the Iranian context underscores its reliability and relevance for dietary analysis in this study.

###  Diet Quality Assessment

 Diet quality was assessed using the HEI-2015 and the DII. Both the HEI-2015 and the DII have been previously validated and applied in Iranian populations. HEI-2015 has shown significant associations with metabolic syndrome components and cardiometabolic markers in Iranian cohorts.^19,20^ Similarly, the DII has been culturally adapted using 30–34 food parameters relevant to Iranian diets, and its construct validity has been supported by studies linking it to inflammation, lipid levels, and diabetes outcomes in Iranian adults.^21,22^

####  Healthy Eating Index-2015 

 Scoring was based on the method by Krebs-Smith et al,^23^ evaluating 13 components divided into adequacy (e.g., whole fruit, vegetables, whole grains, legumes, dairy, protein sources, fatty acids) and moderation (refined grains, sodium, added sugars, saturated fats). Individuals in the highest decile for whole grains, fatty acid ratio, and dairy received a score of 10, while those in the lowest decile received 0. Conversely, those with the highest intake of refined grains, added sugars, sodium, and saturated fats scored 0, while the lowest intake earned a 10. For whole fruit, total fruit, vegetables, legumes, protein foods, seafood, and plant protein, the highest quintile scored 5, and the lowest scored 0. The total HEI-2015 score, ranging from 0 to 100, was calculated by summing all component scores.

####  Dietary Inflammatory Index 

 DII scores were calculated using two-day food records and 24-hour recall questionnaires, following Shivappa et al’s method.^24^ This approach assesses 45 foods and nutrients based on their pro- or anti-inflammatory effects ( + 1, -1, or 0). To align with Iranian diets, we used 30 relevant food parameters, excluding certain items like polyphenols due to data limitations. Nutrient values were energy-adjusted using the residual method and standardized against global means from 11 international datasets. These values were converted into percentile scores, weighted by their inflammatory impact, and summed to derive each participant’s DII score. Higher DII scores indicate a more inflammatory diet, while lower scores suggest an anti-inflammatory diet.

###  Anthropometric Measurements

 Height was measured to the nearest millimeter using a fixed tape, while weight was recorded to with an error of 0.1 kg with a Seca scale. Body mass index (BMI) was calculated by dividing the square of the person’s height (in meters) by their weight (in kilograms). Waist circumference was measured according to National Institutes of Health standards.^25^

###  Statistical Analysis

 Statistical analyses were performed using SPSS software (version 16.0, Chicago, IL, USA). Baseline characteristics were summarized as means ± standard deviations or medians (interquartile ranges) for continuous variables and percentages for categorical variables. Data normality was assessed using the Kolmogorov–Smirnov test. Paired t-tests and Wilcoxon signed-rank tests were used to assess within-subject differences over time. The McNemar test was used for paired categorical variables.

 Changes in HEI-2015 and DII scores were calculated by subtracting baseline values from follow-up values. Participants were categorized into tertiles based on the magnitude of change, and logistic regression models were used to estimate odds ratios (ORs) and 95% confidence intervals (CIs) for associations between dietary changes and metabolic outcomes (e.g., elevated LDL, FBS, or TG). Models were adjusted for age, sex, BMI, waist circumference, and energy intake. Subgroup analyses by age, sex, and BMI category were performed for exploratory purposes. Given the limited sample size, the number of covariates in adjusted models was minimized to reduce the risk of overfitting. Statistical significance was set at *P* < 0.05.

 Only participants with complete dietary, anthropometric, and biochemical data at both time points were included (n = 103). As such, no imputation for missing data was required, and a complete-case analysis was performed. We acknowledge this may introduce selection bias and have addressed it as a study limitation.

 The final sample of 103 individuals was determined by the number of participants with complete longitudinal data from the Azar cohort. Although the sample size was not based on an a priori power calculation, it represents the total eligible cohort available for analysis. To assess the adequacy of this sample size, we conducted a post hoc power analysis using G*Power software (version 3.1). Assuming a two-sided logistic regression model, an alpha level of 0.05, 30% outcome prevalence (e.g., elevated LDL), and an expected odds ratio of 0.5 between tertile extremes, the statistical power was calculated to be approximately 62%. These results indicate that the study was moderately powered to detect large effect sizes but underpowered for smaller associations. Binary metabolic outcomes were defined based on the clinical thresholds recommended by the Adult Treatment Panel III (ATP III) guidelines. General obesity was defined as a BMI ≥ 30 kg/m^2^. Abdominal obesity was identified using waist circumference thresholds of ≥ 102 cm for men and ≥ 88 cm for women. Hyperglycemia was defined as a FBS level ≥ 126 mg/dL. Hypertriglyceridemia was defined as serum TG ≥ 150 mg/dL. Low HDL-C was defined as < 40 mg/dL in men and < 50 mg/dL in women. High LDL-C was defined as ≥ 130 mg/dL. Each of these outcomes was coded as a binary variable (yes/no) and used as a dependent variable in logistic regression analyses.

## Result

###  Baseline Characteristics

 A total of 103 participants were included in the study, with 55.8% male and 44.2% female. The mean age at baseline was 53.6 years. Compared to follow-up, participants had higher body weight and waist circumference at baseline, while BMI was slightly lower.

 At follow-up, participants showed significantly reduced waist circumference (mean difference = 1.33 cm, *P* = 0.01) and TG (median difference = 27 mg/dL, *P* < 0.001). However, changes in BMI (*P* = 0.982) and FBS (*P* = 0.063) were not statistically significant. Smoking and alcohol consumption declined slightly, but McNemar’s test did not show significant changes (smoking: *P* = 0.219; alcohol: *P* = 0.344) (Table 1). Diet quality scores declined slightly over the study period. The mean HEI-2015 score decreased by 4.29 points (from 60.52 ± [8.16] to 56.22 ± [9.64]; *P* < 0.001), while the DII score showed a minimal change (from −3.09 ± 0.92 to −3.05 ± 1.09; *P* = 0.73). Significant differences in HEI-2015 component scores were observed across tertiles at both time points, particularly for whole grains (*P* < 0.001), dairy (*P* < 0.001), seafood and plant proteins (*P* < 0.001), and fatty acid ratios (*P* < 0.001) based on Kruskal-Wallis and ANOVA tests (Table 2). At baseline, participants in the highest HEI tertile had significantly higher scores in most components—except for whole fruits (*P* = 0.19). During follow-up, this group maintained higher intake scores for greens and beans (*P* < 0.01), protein foods (*P* = 0.02), refined grains (*P* < 0.001), sodium (*P* < 0.001), and added sugars (*P* = 0.04), but had significantly lower scores for whole grains (*P* < 0.001), dairy (*P* < 0.001), and unsaturated-to-saturated fatty acid ratio (*P* < 0.001). Changes in total fruit and fatty acid ratio scores also differed significantly across tertiles (*P* < 0.05).

**Table 1:** Baseline characteristics of study participants

**Variables **	**Baseline (n=103) **	**Study Endpoint (n=103) **	***P*** value^*^
	**Mean±SD**	**Mean±SD**	
Age (year)	53.60 ± 7.60	60.35 ± 7.97	**<0.001**
Weight (kg)	81.27 ± 14.10	80.5 ± 13.14	0.091**
Median (interquartile range)	79.95(18)	77.65(16.02)	
BMI (kg/m^2^)	30.10 ± 4.83	30.31 ± 4.53	0.529**
Median (interquartile range)	29.12 (5.93)	29.62(5.85)	
Waist circumference (cm)	101.40 ± 11.28	100.07 ± 10.22	**0.014**
FBS (mg/dL)	137.07 ± 62.96	145.42 ± 60.51	0.063**
Median (interquartile range)	115(65)	128(51)	
TG (mg /dL)	181.92 ± 99.16	140.30 ± 60.97	< 0.001**
Median (interquartile range)	153(95)	126(55)	
Cholesterol (mg /dL)	188.10 ± 43.97	165.35 ± 41.09	<0.001**
Median (interquartile range)	181(52)	164(49)	
LDL (mg /dL)	108.98 ± 34.80	102.62 ± 36.05	0.132**
Median (interquartile range)	105(45)	97(43)	
High-density lipoprotein (mg /dL)	42.72 ± 10.20	35.56 ± 6.36	<0.001**
Median (interquartile range)	41(14)	34(8)	
	**No. (%)**	**No. (%)**	
Smoker	16(15.38)	12 (11.53)	0.219***
Alcohol consumption	11(10.57)	7(6.73)	0.344***
Gender (Male %) **#	(55.8)		
Marital status (Married) (n) **#	(100)		
Educational level (n) **#			
Under diploma	Primary school (0)/ secondary school (43)		
Diploma	(47)		
Higher education	(13)		

*P values by paired t-tests; ^**^P value byWilcoxon test; ^***^P value by McNemar test; **#**Categorical variables were presented for baseline only to simplify the presentation and avoid redundancy. SD: standard deviation; BMI, Body mass index; FBS, fasting blood sugar; TG, Triglycerides; LDL, Low-density lipoprotein; HDL, high-density lipoprotein.

**Table 2:** Comparison of Healthy Eating Index-2015 Total Scores, Dietary Inflammatory Index Scores, and HEI Component Scores Across Tertiles

**Variables **	**Min-max score**	**Baseline **					**Study Endpoint**					
		**Total**	**T1**	**T2**	**T3**	**CI**	**Total**	**T1**	**T2**	**T3**	**CI**	**Diff**
Adequacy												
Total fruits score	0-5	4.84 *	4.77	4.92	4.84	(4.73, 4.95)	4.73 *	4.38	4.94	4.88	(4.59, 4.87)	0.10
Whole Fruits score	0-5	4.99 *	5.00	4.99	4.99	(4.99, 5.00)	4.96 *	4.88	5.00	5.00	(4.91, 5.00)	0.03
Total vegetable score	0-5	4.92 *	4.87	4.93	4.97	(4.87. 4.97)	4.94 *	4.93	4.93	4.97	(4.90, 4.99)	-0.02
Greens and beans score	0-5	4.46 *	4.05	4.54	4.80	(4.29, 4.64)	4.60 *	4.21	4.78	4.82	(4.43, 4.77)	-0.13
Whole grains score	0-10	2.66 **	1.16	1.72	5.05	(2.00, 3.31)	3.18 **	6.71	1.96	0.98	(2.42, 3.81)	-0.52
Dairy score	0-10	7.12 *	6.18	7.43	7.77	(6.70, 7.54)	3.93 **	4.56	3.61	3.66	(3.52, 4.38)	3.18
Total protein foods score	0-5	2.87 *	2.40	2.88	3.33	(2.68, 3.06)	2.87 *	2.27	3.01	3.32	(2.66, 3.07)	0.002
Seafood and plant proteins score	0-5	3.52 *	2.78	3.75	4.03	(3.29, 3.75)	1.61 *	1.16	1.84	1.85	(1.46, 1.77)	1.90
Unsaturated to saturated fatty acids ratio score	0-10	0.8 **	0.25	0.80	1.42	(0.57, 1.08)	1.69 **	2.79	1.65	0.65	(1.26, 2.05)	-0.86
Total	0-60	36.25 *	31.49	36.01	41.24	(35.23, 37.27)	32.55 *	27.16	31.75	38.93	(31.40, 33.71)	3.69
Moderation												
Refined grains score	0-10	1.41**	0.43	0.91	2.87	(0.87, 1.95)	1.99 **	0.17	0.74	5.14	(1.32, 2.65)	-0.57
Sodium score	0-10	6.93 *	5.41	7.40	7.99	(6.21, 7.64)	5.75 *	4.22	6.65	6.21	(5.28, 6.23)	1.17
Added sugars score	0-10	9.16 *	8.76	9.31	9.43	(8.83, 9.50)	9.28 *	9.11	9.23	9.50	(9.06, 9,50)	-0.09
Saturated fats score	0-10	6.75 *	5.82	6.72	7.70	(6.19, 7.30)	6.72 *	5.60	7.40	7.17	(6.18, 7.25)	0.03
Total	0-40	24.26 *	20.43	24.35	28.01	(23.32, 25.21)	23.66 *	19.11	23.77	28.23	(22.63, 24.68)	0.60
Total HEI Score	**0-100**	**60.52**	51.92	60.36	69.25	(46.28, 74.91)	**56.22**	46.27	55.52	67.16	(34.64, 58.30)	4.29
Total DII Score	**–8.87, -7.98**	**-3.09±0.92**	-2.61	-3.15	-3.52	(-4.32, -2.03)	**-3.05±1.09**	-2.42	-3.38	-3.37	(-4.20, -1.91)	-0.04

* One-way ANOVA test, ** Kruskal-Wallis test, T: tertiles, CI: confidence interval, HEI: healthy eating index, DII: dietary inflammatory index; diff: differences between “total” columns. Note: Values are presented as mean ± SD. CI indicates the 95% confidence interval for the total score. Bold numbers represent the overall scores derived from the two dietary indices.

###  Relationship Between HEI-2015 Score Difference and Study Outcomes

 The association between changes in HEI-2015 scores and metabolic outcomes is presented in Table 3. At follow-up, 69 participants (32.2%) had general obesity (BMI ≥ 30 kg/m^2^), and 91 (42.5%) had FBS levels indicative of hyperglycemia (FBS ≥ 126 mg/dL). Additionally, 122 individuals (57.0%) had low HDL-C, 86 (40.2%) had high TG (TG ≥ 150 mg/dL), and 73 (34.1%) had high LDL-C (LDL ≥ 130 mg/dL). Across all models, higher HEI-2015 tertiles tended to show reduced odds of elevated glycemic and LDL levels, although these associations did not reach statistical significance. Conversely, trends toward increased HDL and cholesterol levels were observed, but again, without statistical significance.

**Table 3:** Association between Healthy eating index-2015 difference at start and reassessment phase and risk of metabolic parameters

**Variables **					
	** T1**	***P*** ** value**	** T2**	***P*** ** value**	** T3**
**Glycemic control (Hyperglycemia: FBS≥126 mg/dL)**					
Overall	** OR (95%)**		** OR (95%)**		
Crude Model	1.13 (0.43, 0.94)	0.798	0.72 (0.27, 1.89)	0.501	Ref
Model 1^a^	1.09 (0.41, 2.86)	0.862	0.68 (0.25, 1.83)	0.452	Ref
Model 2^b^	1.08 (0.40, 2.88)	0.871	0.67 (0.24, 1.85)	0.440	Ref
Subgroup by age					
Crude model	** OR (95%)**		** OR (95%)**		
> 60	1.28 (0.32, 5.16)	0.727	0.69 (0.18, 2.53)	0.571	Ref
≤ 60	1.03 (0.27, 3.91)	0.950	0.76 (0.17, 3.24)	0.71 6	Ref
Model 1^c^					
> 60	1 (0.23, 4.32)	0.999	0.61 (0.16, 2.35)	0.472	Ref
≤ 60	1.08 (0.28, 4.12)	0.903	0.78 (0.18, 3.34)	0.737	Ref
Model 2^d^					
> 60	0.93 (0.20, 4.19)	0.926	0.50 (0.12, 2.10)	0.355	Ref
< 60	1.28 (0.31, 5.20)	0.726	0.86 (0.19, 3.89)	0.850	Ref
Subgroup by BMI					
Crude model	** OR (95%)**		** OR (95%)**		
≥ 30	0.97 (0.27, 3.51)	0.977	0.54 (0.12, 2.36)	0.419	Ref
< 30	1.25 (0.28, 5.40)	0.762	0.76 (0.19, 3.12)	0.716	Ref
Model 1^a^					
≥ 30	0.93 (0.25, 3.40)	0.917	0.55 (0.12, 2.43)	0.432	Ref
< 30	1.25 (0.28, 5.56)	0.769	0.68 (0.16, 2.90)	0.611	Ref
Model 2^e^					
≥ 30	1.08 (0.27, 4.24)	0.907	0.58 (0.13, 2.61)	0.487	Ref
< 30	1.12 (0.24, 5.22)	0.889	0.68 (0.15, 3.06)	0.615	Ref
Subgroup by gender					
Crude model	** OR (95%)**		** OR (95%)**		
Male	0.95 (0.25, 3.51)	0.948	0.58 (0.15, 2.25)	0.433	Ref
Female	1.22 (0.28, 5.25)	0.784	0.85 (0.21, 3.47)	0.826	Ref
Model 1^f^					
Male	0.94 (0.25, 3.5)	0.932	0.54 (0.13, 2.15)	0.385	Ref
Female	1.19 (0.27, 5.20)	0.816	0.88 (0.21, 3.68)	0.869	Ref
Model 2^g^					
Male	0.60 (0.15, 2.45)	0.480	0.94 (0.25, 3.53)	0.937	Ref
Female	0.66 (0.14, 3.04)	0.593	1.32 (0.28, 6.14)	0.712	Ref
**LDL (high LDL:≥130 mg/dL)**					
Overall	** OR (95%)**		** OR (95%)**		
Crude Model	1.00 (0.38, 2.59)	1.000	0.89 (0.34, 2.32)	0.816	Ref
Model 1^a^	1.02 (0.38, 2.71)	0.964	1.00 (0.37, 2.65)	0.999	Ref
Model 2^b^	0.93 (0.34, 2.56)	0.908	0.87 (0.31, 2.42)	0.801	Ref
Subgroup by age					
Crude model					
> 60	0.75 (0.18, 3.05)	0.682	0.69 (0.18, 2.53)	0.572	Ref
≤ 60	1.16 (0.30, 4.42)	0.824	1.31 (0.30, 5.58)	0.713	Ref
Model 1^c^					
> 60	0.80 (0.19, 3.36)	0.763	0.70 (0.19, 2.61)	0.608	Ref
≤ 60	1.22 (0.31, 4.71)	0.762	1.35 (0.31, 5.84)	0.684	Ref
Model 2^d^					
> 60	0.64 (0.13, 3.09)	0.571	0.38 (0.08, 1.76)	0.211	Ref
< 60	1.20 (0.30, 4.78)	0.799	1.57 (0.35, 7.09)	0.559	Ref
Subgroup by BMI					
Crude model					
≥ 30	0.63 (0.17, 2.31)	0.492	0.62 (0.15, 2.58)	0.518	Ref
< 30	1.57 (0.35, 6.87)	0.544	1.09 (0.27, 4.40)	0.900	Ref
Model 1^a^					
≥ 30	0.88 (0.80, 0.96)	0.009	0.64 (0.13, 3.04)	0.578	Ref
< 30	1.83 (0.40, 8.42)	0.437	0.98 (0.23, 4.23)	0.985	Ref
Model 2^e^					
≥ 30	0.45 (0.09, 2.14)	0.320	0.64 (0.13, 3.07)	0.583	Ref
< 30	1.75 (0.36, 8.41)	0.484	1.04 (0.23, 4.78)	0.953	Ref
Subgroup by gender					
Crude model					
Male	1.63 (0.43, 6.11)	0.461	1.00 (0.25, 3.92)	1.000	Ref
Female	0.60 (0.14, 2.57)	0.495	0.90 (0.22, 3.58)	0.889	Ref
Model 1^f^					
Male	1.65 (0.43, 6.31)	0.456	1.13 (0.28, 4.55)	0.862	Ref
Female	0.54 (0.12, 2.42)	0.431	0.95 (0.23, 3.88)	0.950	Ref
Model 2^g^					
Male	1.56 (0.38, 6.44)	0.538	1.27 (0.28, 5.81)	0.755	Ref
Female	0.38 (0.07, 1.97)	0.251	0.88 (0.17, 4.42)	0.889	Ref
**HDL (low HDL:<40 mg/dL in men,<50 mg/dL in women)**					
**Overall **	** OR (95%)**		** OR (95%)**		
Crude Model	0.82 (0.17, 4.01)	0.810	1.10 (0.2, 5.90)	0.905	Ref
Model 1^a^	1.08 (0.21, 5.61)	0.928	0.99 (0.17, 5.67)	0.999	Ref
Model 2^b^	1.13 (0.21, 6.07)	0.889	0.96 (0.16, 5.76)	0.973	Ref
Subgroup by age					
Crude model	** OR (95%)**		** OR (95%)**		
> 60	1.86 (0.15, 22.93)	0.624	1.26 (0.15, 10.07)	0.820	Ref
≤ 60	0.42 (0.04, 4.57)	0.489	0.92 (0.05, 16.42)	0.963	Ref
Model 1^c^					
> 60	1.95 (0.15, 25.2)	0.605	1.28 (0.16, 10.35)	0.817	Ref
≤ 60	0.5 (0.04, 6.01)	0.589	1.00 (0.05, 19.96)	1.000	Ref
Model 2^d^					
> 60	1.76 (0.13, 23.2)	0.669	0.86 (0.09, 7.81)	0.898	Ref
< 60	0.65 (0.04, 9.46)	0.750	1.31 (0.05, 33.79)	0.862	Ref
Subgroup by BMI					
Crude model	** OR (95%)**		** OR (95%)**		
≥ 30	0.97 (0.28, 3.55)	0.900	0.34 (0.18, 2.96)	0.417	Ref
< 30	1.25 (0.28, 5.40)	0.703	0.71 (0.29, 4.10)	0.712	Ref
Model 1^a^					
≥ 30	1.01 (0.21, 7.10)	1.000	0.87 (0.18, 3.89)	0.561	Ref
< 30	1.95 (0.57, 8.03)	0.312	1.27 (0.20, 6.69)	0.619	Ref
Model 2^e^					
≥ 30	1.03 (0.09, 13.22)	0.800	0.65 (0.05, 8.20)	0.600	Ref
< 30	0.70 (0.08, 5.11)	0.558	1.04 (0.12, 8.40)	0.715	Ref
Subgroup by gender					
Crude model	** OR (95%)**		** OR (95%)**		
Male	0.33 (0.03, 3.37)	0.354	0.38 (0.03, 4.09)	0.424	Ref
Female	0.45 (0.18, 9.01)	0.329	0.23 (0.19, 7.34)	0.216	Ref
Model 1^f^					
Male	0.37 (0.03, 4.03)	0.427	0.27 (0.02, 3.13)	0.299	Ref
Female	0.24 (0.02, 3.65)	0.333	0.21 (0.01, 2.98)	0.552	Ref
Model 2^g^					
Male	0.36 (0.03, 4.34)	0.424	0.26 (0.02, 3.35)	0.304	Ref
Female	0.25 (0.01, 4.33)	0.372	0.19 (0.01, 3.18)	0.333	Ref
**TG: (high TG≥150 mg/dL)**					
**Overall **	** OR (95%)**		** OR (95%)**		
Crude Model	1.44 (0.51, 4.03)	0.489	1.12 (0.39, 3.20)	0.824	Ref
Model 1^a^	1.29 (0.43, 3.82)	0.642	1.41 (0.47, 4.21)	0.539	Ref
Model 2^b^	1.30 (0.42, 3.95)	0.642	1.40 (0.44, 4.40)	0.561	Ref
Subgroup by age					
Crude model	** OR (95%)**		** OR (95%)**		
> 60	4.00 (0.36, 43.38)	0.256	6.40 (0.68, 59.58)	0.114	Ref
≤ 60	0.79 (0.21, 3.00)	0.737	0.43 (0.10, 1.91)	0.274	Ref
Model 1^c^					
> 60	4.79 (0.41, 54.87)	0.205	6.95 (0.73, 66.19)	0.096	Ref
≤ 60	0.41 (0.09, 1.84)	0.248	0.74 (0.19, 2.58)	0.661	Ref
Model 2^d^					
> 60	5.07 (0.42, 61.34)	0.200	6.13 (0.59, 62.82)	0.128	Ref
< 60	0.8 (0.2, 3.22)	0.751	0.47 (0.10, 2.16)	0.336	Ref
Subgroup by BMI					
Crude model	** OR (95%)**		** OR (95%)**		
≥ 30	1.00 (0.21, 4.70)	1.000	0.87 (0.19, 3.89)	0.863	Ref
< 30	1.90 (0.47, 7.63)	0.365	1.33 (0.28, 6.30)	0.717	Ref
Model 1^a^					
≥ 30	1.60 (0.37, 6.93)	0.527	1.46 (0.28, 7.44)	0.640	Ref
< 30	1.01 (0.19, 5.37)	0.999	1.21 (0.24, 6.11)	0.815	Ref
Model 2^e^					
≥ 30	1.96 (0.41, 9.32)	0.399	1.62 (0.31, 8.52)	0.561	Ref
< 30	0.86 (0.15, 4.85)	0.879	1.13 (0.2, 6.33)	0.882	Ref
Subgroup by gender					
Crude model	** OR (95%)**		** OR (95%)**		
Male	0.8 (0.2, 3.06)	0.745	0.5 (0.11, 2.12)	0.349	Ref
Female	2.91 (0.54, 15.56)	0.21	2.80 (0.56, 13.95)	0.238	Ref
Model 1^f^					
Male	0.74 (0.17, 3.09)	0.680	0.60 (0.13, 2.72)	0.510	Ref
Female	2.52 (0.44, 14.27)	0.294	3.79 (0.67, 21.36)	0.139	Ref
Model 2^g^					
Male	0.73 (0.17, 3.09)	0.671	0.66 (0.14, 3.03)	0.593	Ref
Female	3.61 (0.5, 25.94)	0.200	2.64 (0.40, 17.44)	0.310	Ref
**Cholesterol (low cholesterol≥200 mg /dL)**					
Overall	** OR (95%)**		** OR (95%)**		
Crude Model	1.08 (0.29, 3.94)	0.900	1.30 (0.36, 4.60)	0.689	Ref
Model 1^a^	1.04 (0.27, 3.93)	0.951	1.55 (0.42, 5.70)	0.502	Ref
Model 2^b^	0.98 (0.24, 3.96)	0.989	1.24 (0.31, 4.97)	0.752	Ref
Subgroup by age					
Crude model	** OR (95%)**		** OR (95%)**		
> 60	0.33 (0.03, 3.60)	0.362	1.09 (0.21, 5.75)	0.910	Ref
≤ 60	1.62 (0.23, 11.46)	0.620	2.03 (0.33, 12.23)	0.438	Ref
Model 1^c^					
> 60	0.35 (0.03, 4.00)	0.403	1.12 (0.21, 5.96)	0.889	Ref
≤ 60	2.07 (0.34, 12.61)	0.426	1.64 (0.23, 11.67)	0.610	Ref
Model 2^d^					
> 60	0.08 (0.002, 3.14)	0.18	0.28 (0.02, 3.02)	0.29	Ref
< 60	2.96 (0.40, 21,62)	0.28	2.04 (0.26, 15.91)	0.49	Ref
Subgroup by BMI					
Crude model	** OR (95%)**		** OR (95%)**		
≥ 30	1.13 (0.19, 6.48)	0.88	0.47 (0.04, 5.10)	0.53	Ref
< 30	1.00 (0.14, 7.09)	1.00	1.76 (0.29, 10.47)	0.53	Ref
Model 1^a^					
≥ 30	0.66 (0.07, 5.84)	0.710	0.51 (0.03, 7.28)	0.622	Ref
< 30	1.10 (0.15, 8.15)	0.923	1.66 (0.27, 10.11)	0.584	Ref
Model 2^e^					
≥ 30	1.03 (0.09, 11.12)	0.980	0.60 (0.04, 8.83)	0.704	Ref
< 30	0.75 (0.09, 6.11)	0.799	1.24 (0.18, 8.42)	0.823	Ref
Subgroup by gender					
Crude model	** OR (95%)**		** OR (95%)**		
Male	2.94 (0.29, 29.32)	0.351	3.50 (0.34, 35.11)	0.285	Ref
Female	0.59 (0.09, 3.86)	0.589	0.75 (0.13, 4.03)	0.730	Ref
Model 1^f^					
Male	2.77 (0.26, 28.67)	0.395	4.43 (0.42, 46.61)	0.216	Ref
Female	0.54 (0.08, 3.65)	0.534	0.80 (0.14, 4.46)	0.807	Ref
Model 2^g^					
Male	4.19 (0.30, 57.72)	0.283	7.65 (0.49, 119.2)	0.148	Ref
Female	0.48 (0.06, 3.72)	0.499	0.47 (0.07, 3.13)	0.442	Ref

TG: triglyceride, HDL: High density lipoprotein, LDL: low density lipoprotein; HEI: healthy eating index. These values are risk ratio (95% CIs) Obtained from logistic regression. a: adjusted for age and gender. b: adjusted for age and gender, BMI and WSI. c: adjusted for gender. d: adjusted for gender. BMI and WSI. e: adjusted for age, gender and WSI. f: adjusted for age, BMI and WSI. g: adjusted for age.

 For the purpose of subgroup analyses, participants were categorized based on age 60 as the cutoff point. Among the total sample, 45 individuals (43.3%) were under 60 years of age, and 58 (55.8%) were aged 60 years or older. This age-based classification was used in stratified models to explore potential effect modification by age. The distribution is reported here to clarify subgroup composition. Subgroup analyses revealed one notable finding. In Model 1, adjusted for age and gender, individuals with a BMI ≥ 30 in the lowest HEI tertile had a significantly lower risk of LDL elevation (OR: 0.88, 95% CI: 0.80–0.96, *P* = 0.009). No other subgroup showed significant associations. These results should be interpreted with caution due to the limited sample size and potential for type I errors from multiple comparisons.

###  Relationship Between DII Score Difference and Study Outcomes

 The analysis of the relationship between changes in DII scores and study outcomes, presented in Table 4, indicated an overall inverse trend: as the DII difference increased, the risk of elevated outcome concentrations declined. However, these associations were not statistically significant in either the crude or adjusted models. Subgroup analyses revealed some isolated significant findings. Among individuals under 60 years old in the highest DII tertile, Model 1 (adjusted for gender and BMI) showed a significant reduction in the risk of LDL elevation (OR: 0.14, 95% CI: 0.02–0.91, *P* = 0.04). Similarly, in individuals with a BMI ≤ 30 within the highest tertile, the same model indicated an 85% reduction in LDL elevation risk (OR: 0.15, 95% CI: 0.02–0.90, *P* = 0.03). Additionally, a subgroup analysis of HDL outcomes by gender in Model 2 (adjusted for age, BMI, and household income) revealed a significant reduction in the risk of HDL elevation among men in the highest tertile (OR: 0.05, 95% CI: 0.004–0.89, *P* = 0.04). No other subgroup analyses yielded significant results. These findings highlight the predominantly null associations between HEI-2015 and DII scores and the primary study outcomes in the overall cohort. The significant results observed in subgroup analyses may reflect specific population dynamics but should be interpreted cautiously due to the small sample size and the increased likelihood of spurious findings from multiple comparisons. Future research with larger cohorts is necessary to validate these observations and explore the underlying mechanisms.

**Table 4:** Association between dietary inflammatory difference at start and reassessment phase and risk of metabolic parameters

**Variables **					
	**T1**	**T2**	***P*** ** value**	**T3**	***P*** ** value**
**Glycemic control (Hyperglycemia: FBS≥126 mg/dL)**					
Overall		**OR (95%)**		**OR (95%)**	
Crude model	Ref	1.50 (0.57, 3.89)	0.407	1.26 (0.48, 3.26)	0.622
Model 1^a^	Ref	1.50 (0.57, 3.93)	0.409	1.25 (0.48, 3.26)	0.636
Model 2^b^	Ref	1.36 (0.51, 3.66)	0.524	1.18 (0.44, 3.14)	0.738
Subgroup by age					
Crude model		**OR (95%)**		**OR (95%)**	
> 60	Ref	1.06 (0.27, 4.09)	0.922	1.37 (0.37, 5.03)	0.630
≤ 60	Ref	2.08 (0.52, 8.23)	0.299	1.16 (0.28, 4.72)	0.823
Model 1^c^					
> 60	Ref	0.92 (0.23, 3.72)	0.915	1.15 (0.30, 4.45)	0.824
≤ 60	Ref	2.00 (0.5, 7.99)	0.323	1.10 (0.26, 4.55)	0.881
Model 2^d^					
> 60	Ref	0.87 (0.21, 3.63)	0.859	1.08 (0.26, 4.49)	0.900
≤ 60	Ref	1.68 (0.40, 6.97)	0.470	0.96 (0.22, 4.09)	0.951
Subgroup by BMI					
Crude model		**OR (95%)**		**OR (95%)**	
≥ 30	Ref	3.85 (0.93, 15.8)	0.060	2.62 (0.62, 11.0)	0.183
< 30	Ref	0.53 (0.13, 2.14)	0.379	0.54 (0.14, 2.12)	0.381
Model 1^a^					
≥ 30	Ref	3.86 (0.88, 16.7)	0.072	2.81 (0.64, 12.21)	0.169
< 30	Ref	0.51 (0.12, 2.10)	0.350	0.52 (0.13, 2.09)	0.364
Model 2^e^					
≥ 30	Ref	4.23 (0.93, 19.1)	0.068	3.23 (0.71, 14.06)	0.126
< 30	Ref	0.39 (0.09, 1.74)	0.227	0.41 (0.09, 1.74)	0.234
Subgroup by gender					
Crude model		**OR (95%)**		**OR (95%)**	
Male	Ref	0.81 (0.22, 2.89)	0.742	0.73 (0.20, 2.89)	0.638
Female	Ref	3.42 (0.75, 15.6)	0.119	2.62 (0.57, 11.9)	0.213
Model 1^f^					
Male	Ref	0.67 (0.17, 2.35)	0.558	0.65 (0.17, 2.49)	0.537
Female	Ref	3.53 (0.71, 17.5)	0.120	2.64 (0.55, 12.5)	0.225
Model 2^g^					
Male	Ref	0.82 (0.22, 2.95)	0.760	0.73 (0.20, 2.57)	0.624
Female	Ref	3.39 (0.73, 15.6)	0.118	2.59 (0.55, 12.04)	0.223
**LDL (high LDL:≥130 mg/dL)**					
Overall		** OR (95%)**		** OR (95%)**	
Crude model	Ref	0.77 (0.29, 2.07)	0.610	0.57 (0.22, 1.51)	0.264
Model 1	Ref	0.65 (0.23, 1.84)	0.428	0.49 (0.17, 1.37)	0.175
Model 2	Ref	0.55 (0.18, 1.62)	0.283	0.41 (0.13, 1.22)	0.114
Subgroup by age					
Crude model		**OR (95%)**		**OR (95%)**	
> 60	Ref	1.18 (0.03, 4.56)	0.800	1.2 (0.32, 4.48)	0.788
≤ 60	Ref	0.31 (0.05, 1.90)	0.201	0.18 (0.03, 1.06)	0.056
Model 1^c^					
> 60	Ref	0.72 (0.16, 3.21)	0.677	0.64 (0.13, 3.01)	0.580
≤ 60	Ref	0.25 (0.03, 1.73)	0.160	0.14 (0.02, 0.91)	**0.041**
Model 2^d^					
> 60	Ref	0.48 (0.09, 2.41)	0.371	0.33 (0.05, 2.11)	0.247
≤ 60	Ref	0.25 (0.03, 1.79)	0.162	0.15 (0.02, 1.02)	0.059
Subgroup by BMI					
Crude model		**OR (95%)**		**OR (95%)**	
≥ 30	Ref	0.89 (0.22, 3.52)	0.871	0.35 (0.09, 1.43)	0.148
< 30	Ref	0.69 (0.16, 2.91)	0.614	0.83 (0.20, 3.42)	0.806
Model 1^a^					
≥ 30	Ref	0.40 (0.07, 2.21)	0.299	0.15 (0.02, 0.90)	**0.031**
< 30	Ref	0.70 (0.16, 2.97)	0.635	0.83 (0.19, 3.48)	0.799
Model 2^e^					
≥ 30	Ref	0.44 (0.07, 2.48)	0.351	0.17 (0.02, 1.05)	0.059
< 30	Ref	0.67 (0.15, 2.90)	0.590	0.81 (0.19, 3.42)	0.778
Subgroup by gender					
Crude model		**OR (95%)**		**OR (95%)**	
Male	Ref	1.09 (0.28, 4.12)	0.899	0.63 (0.17, 2.31)	0.490
Female	Ref	0.51 (0.12, 2.24)	0.382	0.51 (0.12, 2.24)	0.385
Model 1^f^					
Male	Ref	0.87 (0.19, 3.98)	0.860	0.58 (0.13, 2.45)	0.462
Female	Ref	0.47 (0.1, 2.11)	0.325	0.46 (0.1, 2.06)	0.317
Model 2^g^					
Male	Ref	0.80 (0.17, 3.74)	0.773	0.49 (0.1, 2.33)	0.374
Female	Ref	0.35 (0.06, 1.82)	0.218	0.38 (0.07, 1.91)	0.246
**HDL (low HDL:<40 mg/dL in men,<50 mg/dL in women)**					
Overall		** OR (95%)**		** OR (95%)**	
Crude model	Ref	0.64 (0.1, 4.13)	0.641	0.37 (0.06, 2.08)	0.268
Model 1	Ref	0.71 (0.1, 4.82)	0.732	0.35 (0.06, 2.10)	0.250
Model 2	Ref	0.56 (0.07, 4.03)	0.567	0.29 (0.04, 1.85)	0.192
Subgroup by age					
Crude model		** OR (95%)**		** OR (95%)**	
> 60	Ref	0.83 (0.04, 14.82)	0.900	0.27 (0.02, 2.95)	0.281
≤ 60	Ref	0.57 (0.04, 6.99)	0.662	0.53 (0.04, 6.58)	0.624
Model 1^c^					
> 60	Ref	0.82 (0.04, 14.93)	0.891	0.27 (0.02, 2.99)	0.284
≤ 60	Ref	0.43 (0.03, 5.92)	0.538	0.37 (0.02, 5.16)	0.463
Model 2^d^					
> 60	Ref	0.71 (0.03, 14.44)	0.820	0.25 (0.02, 3.25)	0.293
≤ 60	Ref	0.15 (0.007, 3.38)	0.235	0.23 (0.01, 4.63)	0.348
Subgroup by BMI					
Crude model		** OR (95%)**		** OR (95%)**	
≥ 30	Ref	0.22 (0.02, 2.44)	0.220	0.73 (0.04, 12.82)	0.822
< 30	Ref	0.16 (0.01, 1.83)	0.275	0.3 (0.03, 3.10)	0.317
Model 1^a^					
≥ 30	Ref	0.24 (0.02, 2.79)	0.255	0.61 (0.03, 11.30)	0.747
< 30	Ref	0.14 (0.02, 2.37)	0.230	0.25 (0.01, 3.90)	0.326
Model 2^e^					
≥ 30	Ref	0.23 (0.02, 2.76)	0.258	0.64 (0.03, 12.17)	0.772
< 30	Ref	0.11 (0.01, 2,97)	0.213	0.16 (0.008, 3.60)	0.250
Subgroup by gender					
Crude model		** OR (95%)**		** OR (95%)**	
Male	Ref	0.5 (0.04, 6.04)	0.584	0.17 (0.01, 1.68)	0.138
Female	Ref	0.93 (0.05, 16.39)	0.960	0.74 (0.02, 11.16)	0.873
Model 1^f^					
Male	Ref	0.48 (0.03, 6.73)	0.598	0.08 (0.006, 1.16)	0.060
Female	Ref	0.86 (0.04, 15.57)	0.921	0.57 (0.03, 10.98)	0.725
Model 2^g^					
Male	Ref	0.33 (0.01, 5.88)	0.453	0.05 (0.004, 0.89)	**0.047**
Female	Ref	0.35 (0.007, 7.73)	0.601	0.43 (0.01, 8.56)	0.455
**TG: (high TG≥150 mg/dL)**					
Overall		**OR (95%)**		**OR (95%)**	
Crude model	Ref	2.02 (0.72, 5.61)	0.172	1.15 (0.4, 3.31)	0.780
Model 1	Ref	1.85 (0.64, 5.39)	0.255	1.09 (0.36, 3.26)	0.874
Model 2	Ref	1.64 (0.53, 5.05)	0.383	1.01 (0.32, 3.20)	0.981
Subgroup by age					
Crude model		** OR (95%)**		** OR (95%)**	
> 60	Ref	2.42 (0.47, 12.30)	0.282	0.66 (0.09, 4.54)	0.675
≤ 60	Ref	1.66 (0.42, 6.56)	0.461	1.48 (0.36, 5.94)	0.577
Model 1^c^					
> 60	Ref	2.63 (0.5, 13.76)	0.258	0.73 (0.10, 5.09)	0.751
≤ 60	Ref	1.83 (0.45, 7.44)	0.394	1.65 (0.39, 6.88)	0.486
Model 2^d^					
> 60	Ref	2.35 (0.39, 14.10)	0.348	0.49 (0.05, 4.12)	0.512
≤ 60	Ref	1.54 (0.35, 6.78)	0.560	1.79 (0.38, 8.40)	0.454
Subgroup by BMI					
Crude model		** OR (95%)**		** OR (95%)**	
≥ 30	Ref	3.11 (0.71, 13.60)	0.137	2.00 (0.43, 9.27)	0.375
< 30	Ref	1.27 (0.3, 5.32)	0.740	0.66 (0.15, 2.91)	0.594
Model 1^a^					
≥ 30	Ref	2.43 (0.49, 11.91)	0.271	2.2 (0.42, 11.36)	0.340
< 30	Ref	1.52 (0.32, 7.17)	0.598	0.75 (0.15, 3.65)	0.724
Model 2^e^					
≥ 30	Ref	2.49 (0.5, 12.42)	0.265	2.38 (0.45, 12.42)	0.302
< 30	Ref	1.22 (0.24, 6.05)	0.800	0.62 (0.12, 3.20)	0.576
Subgroup by gender					
Crude model		** OR (95%)**		** OR (95%)**	
Male	Ref	1.57 (0.41, 5.95)	0.508	0.72 (0.17, 2.92)	0.647
Female	Ref	2.88 (0.56, 14.68)	0.201	2.16 (0.41, 11.30)	0.355
Model 1^f^					
Male	Ref	1.52 (0.36, 6.35)	0.560	0.80 (0.18, 3.53)	0.779
Female	Ref	2.59 (0.49, 13.55)	0.257	1.87 (0.34, 10.18)	0.463
Model 2^g^					
Male	Ref	1.42 (0.32, 6.22)	0.638	0.71 (0.16, 3.21)	0.660
Female	Ref	3.53 (0.61, 20.3)	0.151	2.07 (0.35, 12.09)	0.415
**Cholesterol (low cholesterol≥200 mg /dL)**					
Overall		**OR (95%)**		**OR (95%)**	
Crude model	Ref	1.30 (0.36, 4.60)	0.687	1.08 (0.29, 3.94)	0.906
Model 1	Ref	1.55 (0.42, 5.70)	0.503	1.04 (0.27, 3.93)	0.952
Model 2	Ref	1.24 (0.31, 4.97)	0.758	0.98 (0.24, 3.99)	0.989
Subgroup by age					
Crude model		** OR (95%)**		** OR (95%)**	
> 60	Ref	1.09 (0.21, 5.75)	0.910	0.33 (0.03, 3.60)	0.367
≤ 60	Ref	1.62 (0.23, 11.46)	0.625	2.03 (0.33, 12.23)	0.433
Model 1^c^					
> 60	Ref	1.12 (0.21, 5.96)	0.887	0.35 (0.03, 4.00)	0.404
≤ 60	Ref	1.64 (0.23, 11.67)	0.610	2.07 (0.34, 12.61)	0.428
Model 2^d^					
> 60	Ref	0.28 (0.02, 3.02)	0.299	0.08 (0.002, 3.14)	0.183
≤ 60	Ref	2.04 (0.26, 15.91)	0.498	2.96 (0.4, 21.62)	0.286
Subgroup by BMI					
Crude model		** OR (95%)**		** OR (95%)**	
≥ 30	Ref	0.47 (0.04, 5.10)	0.532	1.13 (0.19, 6.48)	0.889
< 30	Ref	1.76 (0.29, 10.47)	0.535	1.00 (0.14, 7.09)	1.000
Model 1^a^					
≥ 30	Ref	0.51 (0.03, 7.28)	0.622	0.66 (0.07, 5.84)	0.712
< 30	Ref	1.66 (0.27, 10.11)	0.586	1.10 (0.15, 8.15)	0.921
Model 2^e^					
≥ 30	Ref	0.6 (0.04, 8.83)	0.705	1.03 (0.09, 11.12)	0.988
< 30	Ref	1.24 (0.18, 8.42)	0.823	0.75 (0.09, 6.11)	0.794
Subgroup by gender					
Crude model		** OR (95%)**		** OR (95%)**	
Male	Ref	3.5 (0.34, 35.11)	0.288	2.94 (0.29, 29.32)	0.350
Female	Ref	0.75 (0.13, 4.03)	0.734	0.59 (0.09, 3.86)	0.582
Model 1^f^					
Male	Ref	4.43 (0.42, 46.61)	0.213	2.77 (0.26, 28.67)	0.391
Female	Ref	0.80 (0.14, 4.46)	0.802	0.54 (0.08, 3.65)	0.537
Model 2^g^					
Male	Ref	7.65 (0.49, 119.2)	0.146	4.19 (0.3, 57.72)	0.285
Female	Ref	0.47 (0.07, 3.13)	0.444	0.48 (0.06, 3.72)	0.490

TG: triglyceride, HDL: High density lipoprotein, LDL: low density lipoprotein; DII: dietary inflammatory index. These values are risk ratio (95% CIs) Obtained from logistic regression. a: adjusted for age and gender. b: adjusted for age and gender, BMI and WSI. c: adjusted for gender. d: adjusted for gender. BMI and WSI. e: adjusted for age, gender and WSI. f: adjusted for age, BMI and WSI. g: adjusted for age.

 To assess the adequacy of the sample size, post hoc power analyses were conducted for the primary metabolic outcomes. The study had approximately 99.6% power to detect the observed 13 mg/dL increase in TG (from 115 to 128 mg/dL), indicating strong sensitivity to detect larger effects. In contrast, power to detect the 5 mg/dL increase in FBS (from 137 to 142 mg/dL) and the 6 mg/dL reduction in LDL (from 108 to 102 mg/dL) was limited, at approximately 43.1% and 57.7%, respectively (assuming a standard deviation of 20 mg/dL and α = 0.05). These findings suggest that while the study was adequately powered for large changes, it may have lacked sufficient power to detect modest associations, particularly in smaller subgroups.

## Discussion

 This longitudinal study examined the association between changes in diet quality—measured by HEI-2015 and DII—and lipid and glycemic outcomes in 103 adults with type 2 diabetes. Although no significant associations were found in the overall analysis, several subgroup findings suggest that diet quality may influence metabolic health under specific conditions. These results highlight the complexity of interactions among diet, inflammation, and lipid metabolism, which are modulated by genetic, environmental, and behavioral factors.

 This unexpected trend may reflect adaptive metabolic responses that counterbalance the anticipated rise in LDL levels.^26^ Nutrient composition and interactions suggest that the DII may not fully reflect the nuanced effects of individual dietary components on lipid metabolism.^27^ Moreover, the pharmacological management of type 2 diabetes, including the use of diabetes medication, might interact with dietary components, potentially mitigating the adverse effects of an inflammatory diet on lipid profiles.^28^ Subgroup analyses highlight the clinical relevance of diet quality and inflammation. While overall findings were not statistically significant, trends suggest meaningful associations. Lower LDL levels in individuals under 60 and those with BMI ≤ 30 in the highest DII tertile emphasize the role of anti-inflammatory diets in lipid metabolism. Similarly, reduced HDL elevation risk in men within this tertile suggests gender-specific dietary effects, warranting further investigation. Improved HEI scores were linked to lower LDL levels in obese individuals, underscoring the importance of diet quality in managing dyslipidemia, possibly through enhanced metabolic sensitivity and reduced insulin resistance. Future research should confirm these findings in larger cohorts and explore metabolic and inflammatory pathways influenced by age, BMI, and gender. Consistent with our results, Vahid et al,^11^ found no association between AHEI and DII scores with lipid profiles but reported that adherence to DASH and international diet indices reduced TG and cholesterol. This may be attributed to anti-inflammatory diets rich in omega-3s, antioxidants, and fiber, which improve endothelial function, reduce insulin resistance, and support lipid metabolism.

 These results align with studies like Esposito et al,^29^ demonstrating the benefits of a Mediterranean diet on cardiovascular risk factors, and Ley et al,^28^ showing how diets high in whole grains, fruits, and vegetables reduce risks of metabolic syndrome and type 2 diabetes. However, NHANES data (2005–2018) on 12,440 individuals indicated a nonlinear relationship between DII scores and hyperlipidemia, with higher inflammatory potential initially protective before increasing risk beyond a threshold.^27^ A study on omega-3 fatty acids found they reduce TG and improve lipid and inflammatory markers in diabetic and cardiovascular patients. However, they were also linked to increased LDL levels in some cardiovascular cases, highlighting the complex interplay between dietary components, inflammation, and lipid metabolism.^30^​​ These studies highlight the nuanced relationship between dietary inflammatory potential and lipid profiles, influenced by nutrients, health conditions, and overall lifestyle. While diets with higher inflammatory potential may harm lipid levels, components like omega-3 fatty acids can provide protective effects under specific conditions. This underscores the importance of a balanced diet and further research into the interactions between dietary inflammation, lipid metabolism, and chronic disease risk.

 The small sample size likely contributed to the non-significant results in this study. Similarly, Lin et al’s^31^ study on 106 kidney transplant patients showed heterogeneous findings, with non-significant associations for TC, HDL, and TG, and a higher LDL risk in the high HEI quantile. Ziaee et al^32^ reported that higher HEI-2015 scores in type 2 diabetes patients correlated with increases in TC and LDL. This may reflect shifts toward healthier fats, like mono- and polyunsaturated fats, which can elevate cholesterol levels when overall fat intake rises or caloric consumption exceeds expenditure. High-quality diets, such as the Mediterranean diet, rich in nuts, olive oil, and fatty fish, often lower LDL and raise HDL, improving the lipid profile despite increasing TC. Additionally, pro-inflammatory diets may elevate inflammatory markers, disrupting lipid metabolism and reducing HDL, which is essential for cardiovascular protection. Increased dietary inflammation could therefore lower HDL and heighten cardiovascular risk.

## Strengths and Limitations

 The strengths of this study include its prospective cohort design and five-year follow-up, which allowed for the assessment of long-term dietary impacts on metabolic outcomes. The use of both HEI-2015 and DII provided a comprehensive evaluation of diet quality and inflammatory potential. Additionally, subgroup analyses by age, BMI, and gender offered more granular insights.

 However, several limitations must be acknowledged. The relatively small sample size (n = 103) limited the statistical power, particularly for subgroup comparisons. The reliance on self-reported dietary data may introduce reporting bias. Furthermore, the findings may not be generalizable beyond the regional context of Northwestern Iran, and the null associations observed in the full cohort may reflect small effect sizes or residual confounding. Another important limitation is the modest sample size, which may have affected the statistical power to detect associations—especially in subgroup analyses. Post hoc power analyses demonstrated that the study had high power (~99.6%) to detect the change observed in TG but lower power for LDL (~57.7%) and FBS (~43.1%). This indicates that the non-significant findings, particularly for glycemic outcomes, should be interpreted cautiously, as the study may have been underpowered to detect small-to-moderate effects.

###  Implications and Future Directions

 These findings underscore the importance of personalized dietary recommendations based on individual factors such as age, BMI, and gender. While the overall associations were not statistically significant, subgroup findings suggest potential differential responses to diet quality and inflammatory potential. Larger prospective studies are needed to validate these results and explore underlying mechanisms, including gene-diet and medication-diet interactions. Effective diabetes management should incorporate individualized nutrition strategies alongside pharmacological and lifestyle interventions.

## Conclusion

 This study found no significant overall associations between changes in HEI-2015 or DII scores and lipid or glycemic outcomes among adults with type 2 diabetes. However, subgroup analyses suggest that dietary patterns may influence metabolic health in specific populations. Higher HEI-2015 scores were associated with lower LDL levels in obese individuals, while higher DII scores were inversely related to LDL elevation in younger and lower-BMI individuals. These results highlight the need for personalized dietary strategies and further research using larger, more diverse cohorts.

## Competing Interests

 The authors have nothing to disclose.

## Data Availability Statement

 The data supporting the findings of this study are available from the corresponding author upon reasonable request.

## Ethical Approval

 The studies protocol received ethical approval from the Tabriz University of Medical Sciences Ethics Committee (Approval ID: IR.TBZMED.REC.1400.882). Written informed consent was obtained from all participants or their legal guardians prior to enrollment.
